# Autologous transplantation of adipose-derived stem cells improves functional recovery of skeletal muscle without direct participation in new myofiber formation

**DOI:** 10.1186/s13287-018-0922-1

**Published:** 2018-07-17

**Authors:** Agata Gorecka, Souzan Salemi, Deana Haralampieva, Federica Moalli, Deborah Stroka, Daniel Candinas, Daniel Eberli, Lukas Brügger

**Affiliations:** 1 Department of Clinical Research, Laboratory for Visceral Surgery and Medicine, University of Bern, Murtenstrasse 35, 3008 Bern, Switzerland; 2 Department of Urology, Laboratory for Tissue Engineering and Stem Cell Therapy, University Hospital Zurich, Zurich, Switzerland; 3 University Clinic for Visceral Surgery and Medicine, Bauchzentrum Bern, Inselspital, CH-3010 Bern, Switzerland; 4 Theodor Kocher Institute, University of Bern, Bern, Switzerland; 5 Current address: Division of Immunology, Transplantation and Infectious Diseases, IRCCS San Raffaele Scientific Institute, Milan, Italy

**Keywords:** Adipose-derived stem cells, Crush injury, Skeletal muscle regeneration, Stem cell therapy, Tissue engineering

## Abstract

**Background:**

Skeletal muscle has a remarkable regenerative capacity. However, extensive damage that exceeds the self-regenerative ability of the muscle can lead to irreversible fibrosis, scarring, and significant loss of function. Adipose-derived stem cells (ADSC) are a highly abundant source of progenitor cells that have been previously reported to support the regeneration of various muscle tissues, including striated muscles. The aim of this study was to evaluate the effect of ADSC transplantation on functional skeletal muscle regeneration in an acute injury model.

**Methods:**

Mouse ADSC were isolated from subcutaneous fat tissue and transplanted with a collagen hydrogel into the crushed tibialis anterior muscle of mice. Recovering muscles were analyzed for gene and protein expression by real-time quantitative polymerase chain reaction and immunohistochemistry. The muscle contractility was assessed by myography in an organ bath system.

**Results:**

Intramuscular transplantation of ADSC into crushed tibialis anterior muscle leads to an improved muscle regeneration with ADSC residing in the damaged area. We did not observe ADSC differentiation into new muscle fibers or endothelial cells. However, the ADSC-injected muscles had improved contractility in comparison with the collagen-injected controls 28 days post-transplantation. Additionally, an increase in fiber cross-sectional size and in the number of mature fibers with centralized nuclei was observed.

**Conclusions:**

ADSC transplantation into acute damaged skeletal muscle significantly improves functional muscle tissue regeneration without direct participation in muscle fiber formation. Cellular therapy with ADSC represents a novel approach to promote skeletal muscle regeneration.

**Electronic supplementary material:**

The online version of this article (10.1186/s13287-018-0922-1) contains supplementary material, which is available to authorized users.

## Background

Skeletal muscle is the most common and widely distributed tissue in the human body [1, 2] composing approximately 40% of the total body weight. Structurally, it is built of parallel multinucleated fibers that contract upon stimulation, generating force [3]. The ability of skeletal muscle to regenerate is highly dependent on endogenous paired box protein domain 7 (Pax7)-positive stem cells called satellite cells (SC) [4, 5] which reside under the basal lamina of each muscle fiber. Immediately upon stress or injury, SC are activated and re-enter the cell cycle. A small fraction of SC return to quiescent state; however, most of them migrate to the injury site as muscle precursor cells (MPC). MPC committed to myogenesis downregulate Pax7 expression and at the same time upregulate the transcription factor myogenic differentiation antigen (MyoD). They further differentiate into myoblasts and myofibers, restoring missing muscle mass [6–9]. The mature myofibers express the characteristic structural proteins like myosin heavy chain (MyHC). This regenerative mechanism is very efficient in response to minor injuries; however, upon large damage the deposition of fibrotic tissue can exceed the intrinsic regenerative capacity, leading to impaired muscle flexibility and strength [10, 11]. Although there are surgical treatment options available, in cases with major damage the outcomes are unsatisfactory [12–14].

Several cell-based therapies have been investigated as a possible treatment for severely damaged skeletal muscles [15–18]. Adipose-derived stem cells (ADSC) have received much attention within the last years, as they are an easily accessible source of stem cells for regenerative therapies. ADSC are mesenchymal stem cells (MSC) that can be isolated in large quantities from the perivascular area of subcutaneous fat tissue by liposuction [19–22]. They are a heterogeneous population of cells at different stages of maturation and differentiation and have broad plasticity and differentiation potential similar to MSC isolated from the bone marrow (BM-MSC) [23–27]. Moreover, the adipose tissue has a significantly higher stem cell density than bone marrow (5% versus 0.01%) [28]. Myogenic differentiation in vitro as well as in vivo of the ADSC has been reported so far in several studies [29–31]. Additionally, ADSC have been shown to support tissue regeneration in a paracrine manner by secreting soluble factors promoting cell survival and tissue regeneration [32, 33]. The aim of this study was to evaluate the therapeutic potential of ADSC on the functional recovery of skeletal muscle.

## Methods

### Cell isolation and culture

All the animal experiments were performed according to Federation for Laboratory Animal Science Associations guidelines and approved by the Animal Care Committee of the Canton Bern, Switzerland (study no. BE 88/12).

The ADSC were isolated and cultured as described previously [34]. Briefly, the subcutaneous white fat pads were removed form 7- to 8-week-old female C57BL/6 mice (Envigo, Netherlands). Harvested fat was minced into small pieces and digested in 0.1% collagenase type I (Sigma-Aldrich, Buchs, Switzerland) solution at 37 °C for 1 h. The samples were centrifuged, the supernatant containing adipocytes was discarded, and the stromal vascular fraction pellet was filtered (100 μm) and cultured in a growth medium (Dulbecco’s modified Eagle’s medium (DMEM)/F12, 10% fetal bovine serum (FBS), 1% penicillin-streptomycin (PS); all from Gibco by Life Technologies, Carlsbad, USA). After 24 h, the nonadherent cells were removed. Cells were passaged at 80–90% confluency. ADSC from passage 3 were used in all experiments.

The mouse SC were isolated as described previously [35]. Briefly, the EDL muscle was removed from the recently euthanized C57BL/6 (females, 5 weeks old) and digested in 0.2% collagenase I (Sigma-Aldrich) for 90 min. After digestion, the single myofibers were transferred to matrigel matrix (Corning)-coated plates (1:10 dilution in DMEM) and cultured in high-serum proliferation-inducing medium containing DMEM, 20% FBS, 10% horse serum (HS), 1% PS (Gibco by Life Technologies), 0.5% chicken embryonic extract (CEE; US Biologicals, Salem, MA), and 10 ng/ml basic fibroblast growth factor (bFGF; PeproTech, Rocky Hill, NJ). SC upon activation migrate from the myofibers on the culture dish. Cells were passaged first time after 7 days and for every other passage at 60% confluency. To induce differentiation, SC were placed in a low-serum medium (DMEM, 2% HS, 4% FBS, 0.5% CEE, 1% PS).

For coculture experiments the SC were plated on matrigel-coated dishes with a cell density of 5 × 10^5^ cells/cm^2^ in proliferation medium. After reaching 60% confluency, the cells were serum starved for 24 h and then placed in coculture with/without ADSC seeded on the inserts (0.4 μm; Greiner Bio-One, St. Gallen, Switzerland) in proliferation or differentiation medium. For the ADSC-conditioned (CM-ADSC) medium, SC were cultured in the same medium composition that was changed every 24 h. After 72 h the cells were processed for real-time quantitative polymerase chain reaction (RT-qPCR) and Western blot (WB) analysis, and the SC in differentiation medium were additionally stained for fiber formation with Giemsa solution (Fluka Chemie, Buchs, Switzerland).

### Flow cytometry analysis

Isolated cells were incubated with primary antibodies for 40 min at 4 °C in phosphate-buffered saline (PBS) supplemented with 2% FBS and 2 mM ethylenediaminetetraacetic acid (EDTA). We used the following directly conjugated antibodies: CD29 (R&D Systems), CD44 (BioLegend), CD90.2 (BioLegend), CD31 (BioLegend), CD34 (BioLegend), stem cell antigen 1 (Sca-1; e-Bioscience), PDGFRα (BioLegend), and PDGFRβ (BioLegend). All stainings were controlled with appropriate isotope control antibodies. Analysis was performed on a SORP LSRII (Becton Dickinson) equipped with five lasers and data were collected with FACS DIVA software. Analysis was performed using FlowJo™ 10.0.8 (Treestar, Ashland, OR). All measurements were performed with three biological replicates.

### Immunocytochemistry

Cells were fixed with 4% paraformaldehyde, permeabilized with 0.1% Triton-X and blocked with 5% bovine serum albumin (BSA) for 1 h. Samples were incubated overnight at 4 °C with the primary antibodies CD44 (BD Pharmingen), CD29 (R&D Systems), CD90.2 (BD Pharmingen), CD31 (Abcam, Cambridge, UK), MyHC (in house production), Pax7 (DSHB, Iowa, USA), and MyoD (Santa Cruz Biotechnology, Heidelberg, Germany). Primary antibodies were detected using goat anti-rabbit or goat anti-mouse IgG antibodies conjugated to fluorescein isothiocyanate (FITC) or Streptavidin Alexa 594. Cells were counterstained for F-actin with phalloidin-FITC (Sigma-Aldrich) or phalloidin-Atto 550 and for nuclear staining with DAPI (Sigma-Aldrich).

Cells were visualized using a fluorescence microscope (Nikon Eclipse 800) or confocal laser scanning microscope (Zeiss LSM 710; Carl Zeiss Microscopy, Oberkochen, Germany).

For fiber formation assay the cultured myofibers were fixed with methanol for 7 min and stained with Giemsa solution (1:20) for 1 h at room temperature. The number of myofibers per high-power field was calculated along with the number of nuclei per myofiber.

### Crush injury model generation

For inducing the muscle acute injury we adapted a previously described crush injury model [36, 37] with minor changes. Thirty-nine female C57BL/6 8-week-old mice (Envigo, Netherlands) were administered with buprenorphine (Temgesic*®*, Reckitt Benckiser, Switzerland) prior to surgery, followed by anesthesia with 2% isoflurane (Forene™, Abbvie) exhalation. Both lower limbs were immobilized, and a 1.5-cm longitudinal incision of the skin was made, exposing the tibialis anterior (TA) muscle. The TA was separated from the tibia and underlying extensor digitorum longus (EDL) muscle. The crush injury was induced on both left and right TA muscles via surgical clamps (Fine Instruments, Germany). The muscles were manually clamped twice over their complete lengths. Immediately after the injury, the ADSC mixed with the collagen hydrogel (Rat collagen I; Corning), or collagen only as control, were implanted into randomly assigned crushed muscle. For each mouse, one TA was always implanted with ADSC and the other TA from the same animal served as a collagen-treated control. To track the ADSC after implantation, the cells were labeled prior to implantation with PKH26-red fluorescent linker (Sigma-Aldrich) according to the manufacturer’s recommendations. For optical projection tomography (OPT) analysis, the ADSC were labeled with Cell Tracker Orange CMTMR™ dye (Thermo Fisher Scientific) according to the manufacturer’s recommendations. A total of 3 × 10^6^ ADSC in suspension in 50 μl collagen hydrogel were implanted.

Morphological, histological, and functional data were collected at 7, 14, and 28 days postinjury. The contractility of the TA muscles was measured ex vivo in an organ bath system and healthy age- and sex-matched animals served as controls.

### Functional measurements of TA muscles

The isometric contractile measurements were performed with the Radnoti Organ Bath System (AD Instruments) coupled with a square pulse electrical stimulator (Grass model S88) and data acquisition platform (AD instruments Power Lab Data Acquisition System and Lab Chart software, Oxford, UK) as described previously [38]. Briefly, the TA muscles were dissected and fixed in the organ bath chamber filled with 5 ml Krebs Henseleit (Sigma-Aldrich) solution at 25 °C and oxygenated with O_2_/CO_2_ (95%/5%). For a single twitch tension measurement, the muscle was stimulated with a single 0.5-ms square pulse at 40 Hz. The tetanus tension frequency was established from the force-frequency relationship, where the maximal force for the healthy muscles is achieved at 120 Hz. The tetanus tension was induced by stimulation with a pulse train for 300 ms at 40 V Hz. The muscles were rested for 3 min between each series and all the force measurements were normalized to the weight of each individual muscle.

### Analysis of TA muscle regeneration

#### Immunohistochemistry

TA muscles were harvested, snap frozen, and cryosectioned at 7 μm. Sections were fixed with 4% paraformaldehyde, permabilized with 0.1% Triton-X, and blocked with 5% BSA for 1 h. Primary antibody α-actinin (Sigma-Aldrich) was detected using the M.O.M kit (Dako, Glostrup, Denmark). The CD31 (Abcam) and Ki67 (Dako) antibodies after overnight incubation at 4 °C were detected using goat anti-rabbit or donkey anti-goat IgG antibodies conjugated to FITC.

#### Histological analysis

Cryosections were fixed with 4% paraformaldehyde and stained for hematoxylin and eosin using standard procedures. The cross-sections were visualized using a Pannoramic 250 Flash III slide scanner (Sysmex Suisse AG, Horgen, Switzerland) and images were acquired using Pannoramic Control Software (3D Histotech, Budapest, Hungary). Myofibers diameters were determined using a Pannoramic Viewer (3D Histotech).

#### Optical projection tomography

To track ADSC incorporation within the whole TA muscle without physical sectioning we used OPT. The TA muscles were harvested from 16 female C57BL/6 8-week-old mice (Envigo, Netherlands) and embedded in 2% ultrapure low-gelling agarose (Sigma). Samples were dehydrated in 100% methanol (Sigma) overnight at room temperature and then cleared in a BABB solution (benzyl alcohol and benzyl benzoate) for a minimum of 3 h at room temperature. The optical sections were acquired with Optical Projection Tomography Scanner (Bioptonics) and analyzed with Imaris software (BitPlane, Zurich, Switzerland).

#### Protein isolation and Western blotting

Total protein extracts were isolated from cells using RIPA buffer supplemented with protease inhibitor cocktail (P8340 Sigma-Aldrich). The protein concentration was determined with the BCA protein assay (Thermo Fisher Scientific, Ecublens, Switzerland).

A total of 20 μg protein from each sample was separated by SDS-PAGE and transferred to polyvinylidene difluoride (PVDF) membranes (Trans-Blot Turbo, Bio-Rad Laboratories, Cressier, Switzerland). The primary antibodies used were: alpha-actinin (Cell Signaling Technology, Danvers, USA), MyHC (DSHB), MyoD (Santa Cruz Biotechnology), Pax7 (Thermo Fisher), and GAPDH (Cell Signaling). Primary antibodies were detected using anti-rabbit or anti-mouse horseradish peroxidase (HRP)-conjugated IgG (DAKO). Signals were visualized using enhanced chemiluminescence (Western Lightning Plus ECL, Perkin Elmer) and developed with CURIX 60 (AGFA). Quantification of the signal was performed using ImageJ software and all the signals were normalized to glyceraldehyde-3-phosphate dehydrogenase (GAPDH) expression.

#### RT-qPCR

Total RNA was isolated from all mouse samples and cells using the TRIzol reagent-based method following the manufacturer’s protocol (Life Technologies). RNA was reverse-transcribed with the Omniscript RT Kit 200 (Qiagen). RT-qPCR was performed using the ABI PRISM 7900 Sequence Detection System (Applied Biosystems) according to the standard protocols. The following mouse TaqMan (Life Technologies) probes were used: Pax7 (Mn01354484_m1), MyoD (Mm00440387_m1), MyHC (Mm00443013_m1), collagen type I (Coll1; Mm00483888_m1), transforming growth factor (TGF)b1 (Mm01178820_m1), and GAPDH (Mm99999915_g1). Expression levels were normalized to the expression level of the GAPDH housekeeping gene. The experiment was repeated with at least three biological replicates and each sample was analyzed in triplicate.

### Statistical analysis

Statistical tests were performed using GraphPad Prism 6 software (GraphPad Prism Software Inc.). Student’s *t* tests were performed for RT-qPCR analysis and WB quantification. For the organ bath analysis, one-way analysis of variance (ANOVA) with Bonferroni correction and paired *t* test were performed. For the histological analysis of the fiber size distribution, two-way ANOVA with multiple comparisons and Sidak corrections were performed. *p* < 0.05 was considered statistically significant. Error bars represent the mean ± standard deviation (SD).

## Results

### Characterization of mouse ADSC and SC

ADSC were isolated and cultured until passage 3 as a morphologically homogenous population (Additional file 1: Figure S1A). Greater than 95% of the population expressed the MSC-specific surface markers CD29, CD44, and CD90, and were negative for hematopoietic stem cell CD34 and endothelial surface marker CD31 (Additional file 1: Figure S1B). Immunocytochemistry confirmed the surface expression of CD29, CD44, and CD90 markers, as well as negative expression for CD31 (Additional file 1: Figure S1C). Expression of all markers was consistent throughout the in-vitro and in-vivo experiments. The primary mouse SC cultured until passage 3 in the high-serum proliferation medium exhibited a uniform, undifferentiated morphology (Additional file 2: Figure S2A) and were positive for Pax7 transcription factor (Additional file 2: Figure S2B). The myogenic potential of the isolated SC was confirmed by transferring the cells into differentiation medium, where a subset of cells upregulated the MyoD transcription factor (Additional file 2: Figure S2C) and further formed multinucleated myotubes, positive for the skeletal muscle-specific structural protein MyHC (Additional file 2: Figure S2D–F).

### Effect of ADSC on satellite cell activation and differentiation in vitro

**Fig. 1:**
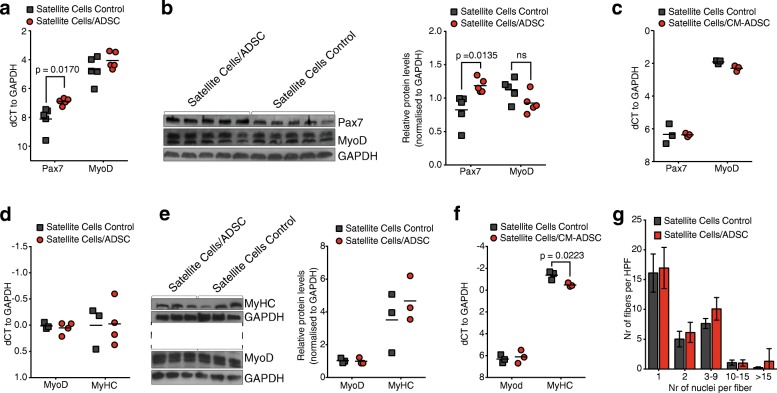
Effect of adipose-derived stem cells (ADSC) on satellite cell (SC) activation and differentiation. **a** Relative mRNA level of paired box protein domain 7 (Pax7) and myogenic differentiation antigen (MyoD) transcription factors in SC cocultured with/without ADSC in proliferation-induction medium. **b** Protein expression of Pax7 and MyoD (upper band) transcription factors in SC cocultured with/without ADSC in proliferation-induction medium. **c** Relative mRNA level of Pax7 and MyoD expressed in SC in proliferation-induction ADSC conditioned medium (CM-ADSC). **d** Relative mRNA level of MyoD and MyHC in MPC cocultured in differentiation-inducing medium with/without ADSC. **e** Relative protein expression of MyoD and MyHC in MPC in differentiation medium cocultured with/without ADSC and relative mRNA levels of expression of MyoD and MyHC in a differentiation-inducing conditioned medium from ADSC. **f** Relative mRNA levels of MyoD and MyHC expressed in MPC in differentiation-induction CM-ADSC. **g** Average number of multinucleated fibers per high-power filed (HPF) between MPC cocultured with/without ADSC, shown as mean ± SD. The relative level of mRNA was measured using RT-qPCR and the relative protein level was determined with Western blot. All the results were normalized to glyceraldehyde-3-phosphate dehydrogenase (GAPDH) expression

To investigate the stimulatory effect of ADSC on SC in vitro, we compared the expression level of myogenic markers between SC directly cocultured with ADSC versus SC cultured in the conditioned medium derived from the ADSC (CM-ADSC). SC directly cocultured with ADSC in proliferative medium after 72 h showed increased mRNA and protein expression of the transcription factor Pax7 (Fig. 1a, b). For SC cultured in proliferative CM-ADSC the same stimulating effect on Pax7 expression was not observed (Fig. 1c). Additionally, in proliferative medium under both culture conditions there were no differences in MyoD expression (Fig. 1a–c). SC cultured with ADSC in differentiation-induction medium also did not show upregulation of MyoD or MyHC expression in both experimental setups of direct coculture and CM-ADSC (Fig. 1d–f). Moreover, cells cultured in CM-ADSC demonstrated a decreased level of MyHC mRNA expression (Fig. 1f). Additionally, for both experimental groups cultured in differentiation-induction medium, the distribution of the average number of nuclei per myotube was similar (Fig. 1g).

### ADSC improve skeletal muscle contractility

**Fig. 2:**
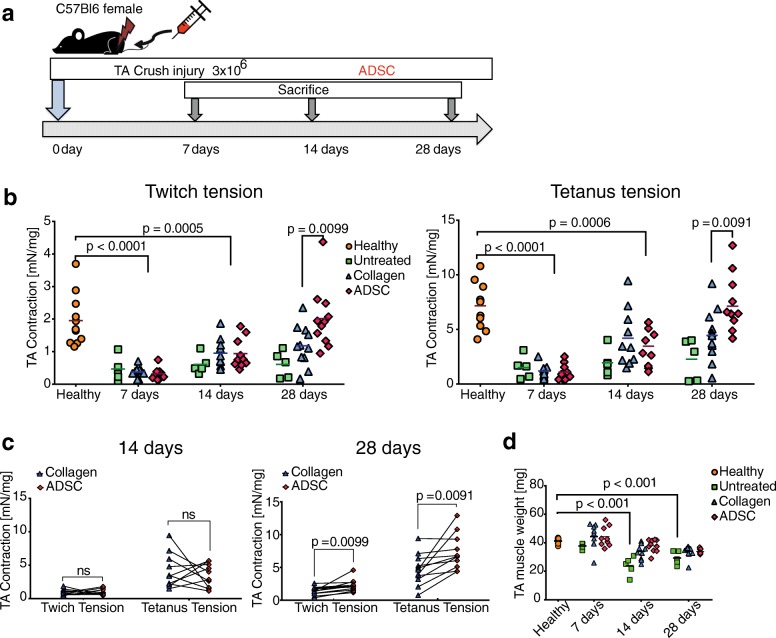
Functional measurements of crushed tibialis anterior (TA) muscles. **a** Experimental design. **b** Organ bath studies of TA muscles after the crush injury. Twitch tension: single 0.5-ms square pulses at 40 Hz. Tetanic tension: series of 0.5 ms pulses for 300 ms at 40 V, 120 Hz. *n* = 10–11 per group. **c** Paired results for the samples measured from the same mice at 14 and 28 days postinjury. Paired *t* test, *n* = 10–11 per group. Results are normalized to the muscle weights. **d** TA average weight at 7, 14, and 28 days postinjury in comparison with the healthy muscle weight; *n* = 5–11 per group

Immediately following a crush injury to the TA muscle, ADSC were implanted and functional analysis in the organ bath system was performed at days 7, 14, and 28 postinjury (Fig. 2a). From the functional analysis, we observed a significant increase in contractility for twitch and tetanus tension at 28 days for the ADSC-treated muscles in comparison with the collagen-treated TAs and muscles that did not receive any treatment (Fig. 2b). Additionally, the strength of the ADSC-treated muscles was similar to the healthy TAs from the healthy age-matched mice. After pairing the muscles from the same animals, ADSC-treated muscles performed better than the collagen controls (Fig. 2c). Moreover, we did not observe any differences in the average muscle weight between the collagen- and cell-treated samples at any of the time points; however, a significant muscle weight reduction in the untreated group was observed at 14 and 28 days postinjury (Fig. 2d).

### ADSC engraft into damaged tissue but do not contribute to skeletal muscle formation in vivo

**Fig. 3:**
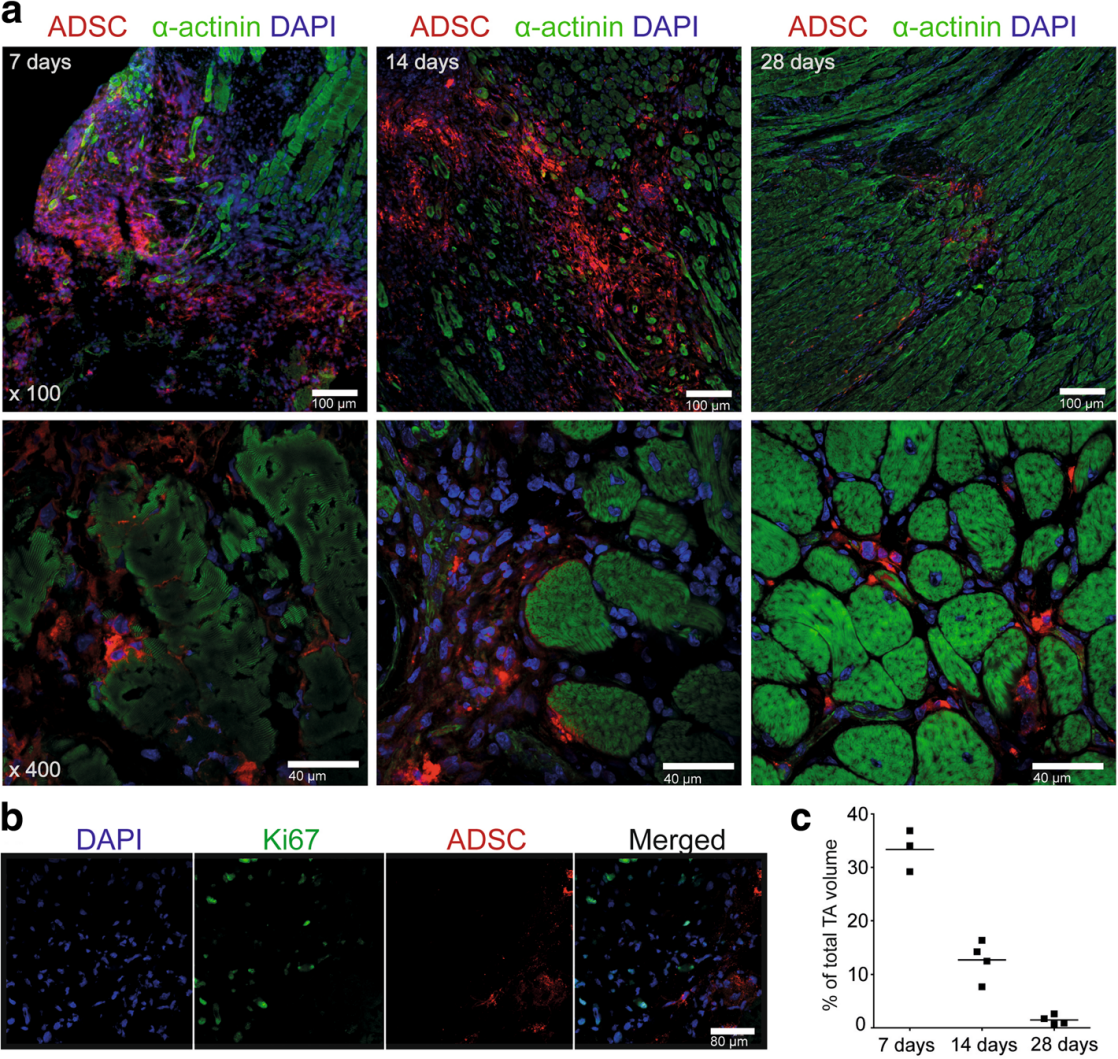
Incorporation of the implanted adipose-derived stem cells (ADSC) into crushed tibialis anterior (TA) muscles. **a** Tracing of the PKH26 red fluorescently labeled ADSC after 7, 14, and 28 days following cell transplantation. Frozen sections of TA muscle were counterstained for α-actinin (green) and cell nuclei (DAPI, blue). Upper panel: scale bars = 100 μm; bottom panel: scale bars = 40 μm. **b** Ki67 staining showing that fluorescently red-labeled ADSC do not proliferate after implantation. Scale bar = 40 μm. **c** Quantification of the ADSC reduction within the TA muscles over time; *n* = 3–4 per group

To elucidate the mechanisms underlying the enhanced contractility of the ADSC-treated muscles, we tracked the implanted cells with OPT microscopy which allowed us to visualize the three-dimensional (3D) pattern of cell distribution within the whole muscle (Additional file 3: Movie S1, Additional file 4: Movie S2, Additional file 5: Movie S3, Additional file 6: Movie S4). At 7 days postinjury, we observed that implanted cells colocalize with the site of the damage, corresponding to 30% of the whole TA volume (Fig. 3c, Additional file 4: Movie S2). At the subsequent time points, we observed a reduction in the number of implanted cells, as well as a decrease in the injury size (Additional file 5: Movie S3, Additional file 6: Movie S4; Additional file 7: Figure S4).

We also detected a fraction of ADSC aligned along healthy myofibers at the border of the injury (Fig. 3a, lower panel). No donor cells were found to be differentiated into myofibers, fused with existing myotubes, or positive for the proliferation marker Ki67 (Fig. 3b) or endothelial marker CD31 (Additional file 8: Figure S3).

### ADSC treatment accelerates myoblast expansion

**Fig. 4:**
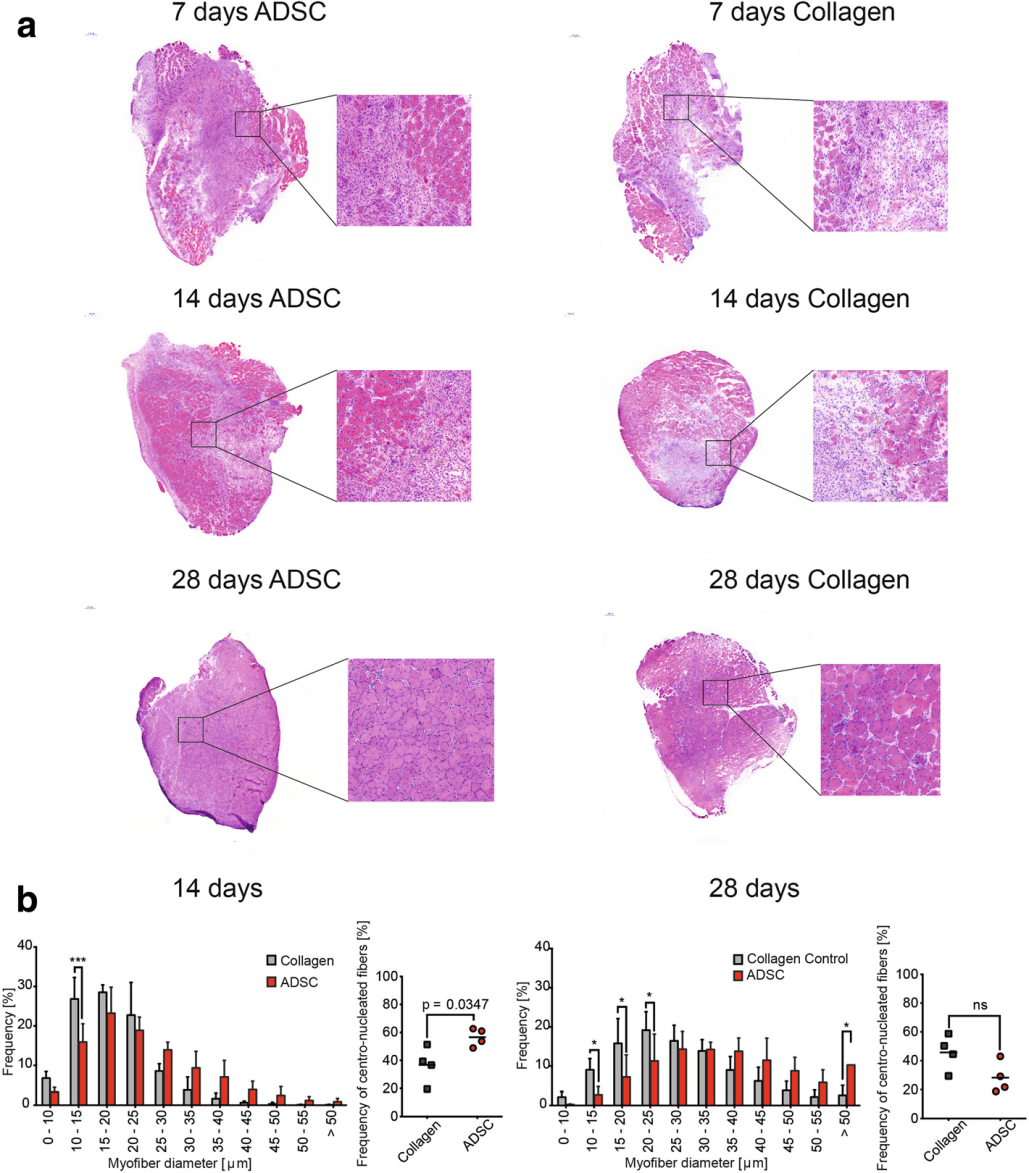
Adipose-derived stem cell (ADSC) treatment positively affects skeletal muscle regeneration. **a** Representative cross sections of TA muscles at 7, 14, and 28 days following crush injury with ADSC or collagen transplantation. **b** Frequency of fiber size distribution for defined ranges of cross-sectional diameter in TA at 14 and 28 days following crush injury with ADSC or collagen transplantation and frequency of centrally nucleated fibers at 14 and 28 days postinjury. Results are shown as mean ± SD from *n* = 3–5 experimental repeats. *p < 0.05, ***p < 0.001

Seven days after the crush injury, most of the damaged area was occupied by mononucleated cells, and sporadic myofibers were present at the periphery of the muscle (Fig. 4). By 14 days postinjury, we observed a higher accumulation of myofibers and a smaller diameter in ADSC-treated muscles. Additionally, we detected a significantly higher number of centrally nucleated immature myofibers in ADSC-treated muscles when compared with the collagen-treated control (Fig. 4b).

At 28 days postinjury, the fraction of large myofibers in the ADSC-treated muscles was significantly higher than in the control samples and, at the same time, the small myofibers were dominant in the control samples. In both experimental groups no fibrotic tissue at 28 days following damage was observed.

### ADSC do not impact myogenesis in early regenerative stages

**Fig. 5:**
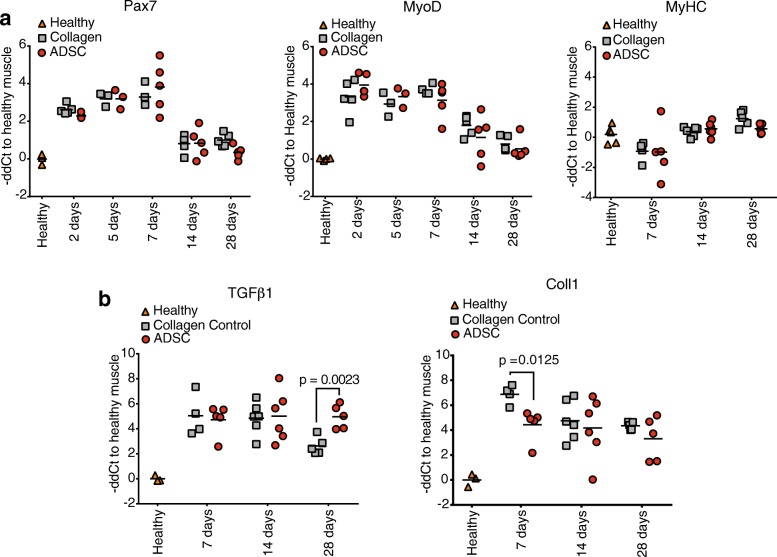
Effect of adipose-derived stem cell (ADSC) transplantation on myogenesis and fibrosis in regenerating TA. **a** Comparative analysis of mRNA from crushed TAs treated with ADSC or collagen hydrogel only. RT-qPCR analysis showed the expression levels of the transcription factors paired box protein domain 7 (Pax7) and myogenic differentiation antigen (MyoD) and structural component myosin heavy chain (MyHC) over time. **b** Comparative level of mRNA measured by RT-qPCR of collagen type I (Coll1) and transforming growth factor β1 (TGFβ1). All results are normalized to GAPDH expression and plotted in relation to healthy TA expression level; *n* = 3–5 per group

Pax7 was upregulated immediately upon injury and peaked between days 5 and 7 following damage (Fig. 5a). This was accompanied by immediate upregulation of MyoD during the first week postinjury, which points to the high myogenesis. The MyHC was downregulated within this time and was restored at later time points. In our study, we did not observe any differences in the expression of these early or late myogenesis markers between the ADSC-treated muscles and collagen-treated controls.

The main component of the muscle extracellular matrix (ECM), collagen type I (Coll1) was upregulated during the entire regenerative process; however, 7 days postinjury we observed higher collagen type I mRNA levels in the collagen-treated controls. This observation did not correlate with TGFβ1 expression (Fig. 5b).

## Discussion

Developing an autologous stem cell therapy for large skeletal muscle injuries would be of great benefit for patients suffering from muscle function loss due to large acute injuries. In regenerative medicine, ADSC are an attractive source of easily accessible MSC with a great regenerative potential. In this study, we demonstrated that a single transplantation of ADSC into the crushed tibialis anterior immediately after the injury significantly accelerates muscle repair in mice by increasing myofiber diameter as well as higher twitch and tetanic force generation. ADSC-treated muscles also showed an earlier appearance of centrally nucleated myofibers, which could explain the greater force generation. Although the possibility of ADSC myogenic transdifferentiation in vivo has been reported [29, 39], in our study we did not observe direct participation of the implanted ADSC in new myofiber formation. This result points towards a possible paracrine modulatory effect of the implanted cells, which would be in line with the growing number of reports describing the supportive properties of ADSC [40–43]. Unfortunately, we were not able to determine the mechanism by which the ADSC are cleared from the injured areas. ADSC could share a similar fate as muscle-resident fibro-adipogenic progenitors (FAPs). It was recently shown that FAPs are activated upon injury to support the first phase of muscle regeneration by paracrine signaling. Once the inflammatory phase of the muscle regeneration is finished, FAPs undergo apoptosis [44–46].

We demonstrated that ADSC have a positive stimulatory effect on SC activation in vitro. Direct coculturing with ADSC induces Pax7 expression in SC when compared with conditioned medium. Therefore, the cross-talk between these two cell populations seems to be essential for SC activation. Additionally, we found that culturing SC in CM-ADSC leads to MyHC downregulation. This observation supports the use of stem cell implantation into the injury site, where the ADSC are expected to have a higher impact on muscle regeneration than delivering only ADSC conditioned medium. This ex-vivo finding is inconsistent with in-vivo studies, where it has been shown that injection of the supernatant is sufficient for achieving a muscle regeneration level comparable to ADSC implantation [47, 48].

From the functional analysis of the harvested muscles, we found that intramuscular transplantation of ADSC into acute injured tibialis anterior muscle helps to restore its contractility within 28 days. There was no indication that the implanted ADSC underwent myogenic or endothelial differentiation, thus suggesting a supportive role of ADSC via paracrine and cell-cell mechanisms. These observations are in line with previous reports, where the implantations of the ADSC into injured muscles have shown a positive effect [49–52]. Additionally, reduction in the weight of the untreated muscles together with very poor functional improvement shows the potential beneficial effect of the collagen hydrogel itself.

One of the major causes of impaired muscle functionality after injury is permanent fibrotic tissue formation. However, in this animal model we did not see connective tissue deposition at the 28-day time point in either experimental groups. This excludes the fibrosis formation as an explanation of the functional discrepancy between the ADSC-treated muscles and the collagen controls. Moreover, the elevated TGFβ1 expression in the ADSC-treated muscles observed at 28 days postinjury was not caused by scar tissue formation. In several studies, TGFβ1 has been described as the most important factor responsible for persistent fibrosis in skeletal muscles [53], but mostly in chronic conditions [54, 55]. In our study, the upregulation of the TGFβ1 could be than associated with the further ECM remodeling; however, the lack of the elevated collagen 1 expression at 28 days postinjury makes this highly unlikely.

Based on the histological assessment of the cross-sectional areas, we concluded that the significant higher force produced in the ADSC-treated group is an effect of a more efficient remodeling and muscle regeneration. It has been proven that the number and the size of the myofibers directly correlates with the strength of the muscle [56], and in our study the frequency distribution for the myofibers size is shifted towards higher number of large and centrally nucleated immature myofibers in the ADSC-treated muscles. The work of Pecanha et al. suggests a similar mechanism, where an injection of ADSC into lacerated soleus rat muscle results in increased force production at 2 weeks postinjury but not at 4 weeks, suggesting that ADSC treatment accelerates muscle recovery [51]. This also implies that we cannot exclude the possibility of the full recovery of the collagen-treated muscles at later time points.

In our in-vivo experimental setup, we did not observe any differences in Pax7 expression between the ADSC-treated muscles and collagen controls. This could be due to the animal model itself, where the crush injury triggers a very high level of SC activation. Other possible explanations are that there is an attenuated effect of the ADSC on SC activation in vivo, or a lack of specificity in our analysis. In our experiment, the RNA was isolated from the site of injury. However, the crush injury activates not only the SC in the injured area, but also SC along the whole myofiber, which might have biased our results.

The last possibility explaining the faster functional recovery of the ADSC-treated muscles is their possible impact on vasculogenesis. Immature myoblasts can function and differentiate in the hypoxic condition that is dominating at the initial phase of the muscle injury. However, for the myoblast fusion and myotube maturation the regrowth of the vasculature is essential [57]. It has been proven in several studies that ADSC improve the vasculature growth and new vessel formation either by differentiation into endothelial cells or by secreting vascular endothelial growth factor (VEGF) [31, 42, 58]. In our crush injury model we did not observe ADSC differentiation into endothelial cells; however, based on the recent published data showing the MSC-mediated vasculature repair of the damaged tissues correlating with increased VEGF levels, we cannot exclude the hypothesis that implanted ADSC support in-vivo muscle regeneration by proangiogenic factor secretion [59–61]. However, this hypothesis needs to be further verified.

## Conclusions

In the present study, we showed that ADSC therapy accelerates the functional recovery of skeletal muscle in mice. This was shown by a significant increase in both twitch and tetanic force and an increase in the number of large, regenerating myofibers. We did not detect any ADSC myogenic differentiation, and therefore we suggest that ADSC act via direct cell-cell or paracrine mechanisms by secreting factors involved in transient acceleration of skeletal muscle repair. Summarizing, ADSC remain attractive candidates for skeletal muscle regeneration therapies; however, further studies are necessary to fully understand their mechanisms of action.

## Additional files

Additional file 1: **Figure S1.** Characterization of mouse primary ADSC. (A) Isolated ADSC at passage 3 present characteristic fibroblast-like morphology. (B) Gating strategy for flow cytometry analysis. (C) Flow cytometry analysis shows that ADSC were positive for the MSC surface markers CD44 (97% ± 2.2), CD90 (95.5% ± 1.5), CD29 (98.6% ± 0.7), and PDGFRβ (57.3% ± 8.8), and negative for the hematopoietic stem cell surface marker CD34 (0.8% ± 0.2) and endothelial surface marker CD31 (1.2% ± 0.4). Data from three independent experiments as mean ± SD. (D) Representative immunocytochemistry confirms the expression of the selected surface markers on the ADSC. (JPG 2826 kb)

Additional file 2: **Figure S2.** Characterization of mouse primary satellite cells. (A) Undifferentiated SC at 60% confluency at passage 3. (B) SC are positive for Pax7 transcription factor. (C) MPC are a fraction of SC committed to myogenesis expressing MyoD transcription factor. (D) Multinucleated myotubes (black arrows) formed by fusion of SC (white arrows) at 7 days in differentiation culture conditions. (E) Fiber formation assay demonstrating long, multinucleated myotubes. Giemsa staining at 5 days in differentiation medium. (F) Myotubes express skeletal muscle-specific myosin heavy chain (MyHC). (JPG 3594 kb)

Additional file 3: **Movie S1.** Control uninjured TA. Optical projection tomography single plane of crushed TAs with implanted ADSC. Blue: myofibers. Red: implanted ADSC. (MP4 387 kb)

Additional file 4: **Movie S2.** TA crush injury 7 days. Optical projection tomography single plane of crushed TAs with implanted ADSC. Blue: myofibers. Red: implanted ADSC. (MP4 700 kb)

Additional file 5: **Movie S3.** TA crush injury 14 days. Optical projection tomography single plane of crushed TAs with implanted ADSC. Blue: myofibers. Red: implanted ADSC. (MP4 470 kb)

Additional file 6: **Movie S4.** TA crush injury 28 days. Optical projection tomography single plane of crushed TAs with implanted ADSC. Blue: myofibers. Red: implanted ADSC. (MP4 382 kb)

Additional file 7: **Figure S4.** OPT of single plane projection of the crushed TAs with implanted ADSC and collagen treated controls at 7, 14, and 28 days postimplantation. Blue: myofibers. Red: implanted ADSC. (JPG 2385 kb)

Additional file 8: **Figure S3.** ADSC do not differentiate into endothelial cells. Representative CD31 (green) staining showing that fluorescently red-labeled ADSC do not overlap with the endothelial cells in the TA muscle. Frozen sections of TA muscle were counterstained for cell nuclei (DAPI, blue). (JPG 5130 kb)

## Abbreviations

ADSC: Adipose-derived stem cells
ANOVA: Analysis of variance
BSA: Bovine serum albumin
CEE: Chicken embryonic extract
CM: Conditioned medium
Coll1: Collagen type 1
DMEM: Dulbecco’s modified Eagle’s medium
ECM: Extracellular matrix
EDL: Extensor digitorum longus
FAP: Fibro-adipogenic progenitor
FBS: Fetal bovine serum
FITC: Fluorescein isothiocyanate
GAPDH: Glyceraldehyde-3-phosphate dehydrogenase
HS: Horse serum
MPC: Muscle precursor cells
MSC: Mesenchymal stem cells
MyHC: Myosin heavy chain
MyoD: Myogenic differentiation antigen
OPT: Optical projection tomography
Pax7: Paired box protein domain 7
PS: Penicillin-streptomycin
RT-qPCR: Real-time quantitative polymerase chain reaction
Sca-1: Stem cell antigen 1
SC: Satellite cells
SD: Standard deviation
TA: Tibialis anterior
TGF: Transforming growth factor
VEGF: Vascular endothelial growth factor
WB: Western blot
