# Supplementary figures and images for: Autologous transplantation of adipose-derived stem cells improves functional recovery of skeletal muscle without direct participation in new myofiber formation
Source: Stem Cell Res Ther. 2018 Jul 17;9:195. doi: 10.1186/s13287-018-0922-1 (PMC6050693; doi:10.1186/s13287-018-0922-1)

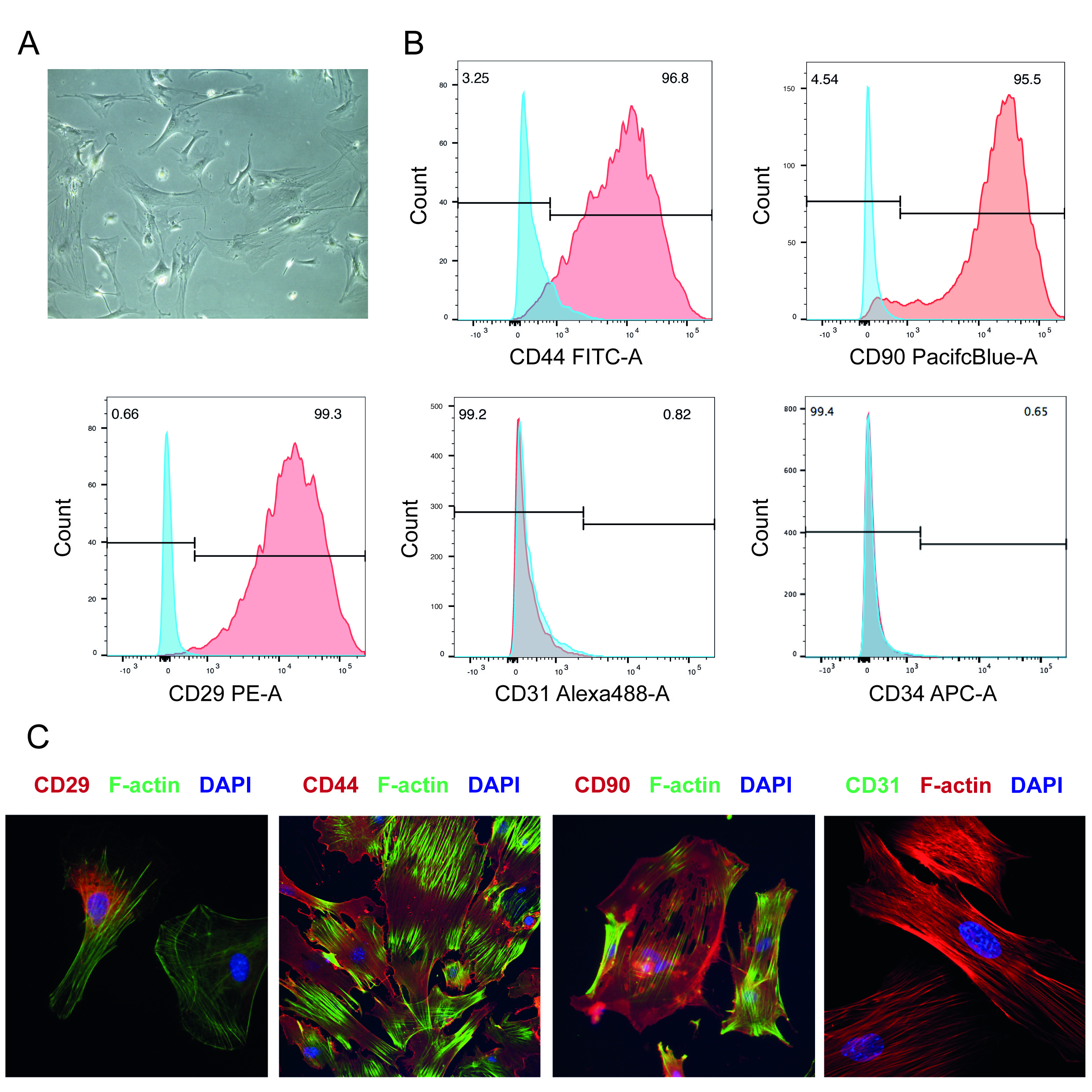

Supplement: Supplementary file 1 — Figure S1. Characterization of mouse primary ADSC. (A) Isolated ADSC at passage 3 present characteristic fibroblast-like morphology. (B) Gating strategy for flow cytometry analysis. (C) Flow cytometry analysis shows that ADSC were positive for the MSC surface markers CD44 (97% ± 2.2), CD90 (95.5% ± 1.5), CD29 (98.6% ± 0.7), and PDGFRβ (57.3% ± 8.8), and negative for the hematopoietic stem cell surface marker CD34 (0.8% ± 0.2) and endothelial surface marker CD31 (1.2% ± 0.4). Data from three independent experiments as mean ± SD. (D) Representative immunocytochemistry confirms the expression of the selected surface markers on the ADSC. (JPG 2826 kb) [file 13287_2018_922_MOESM1_ESM.jpg]

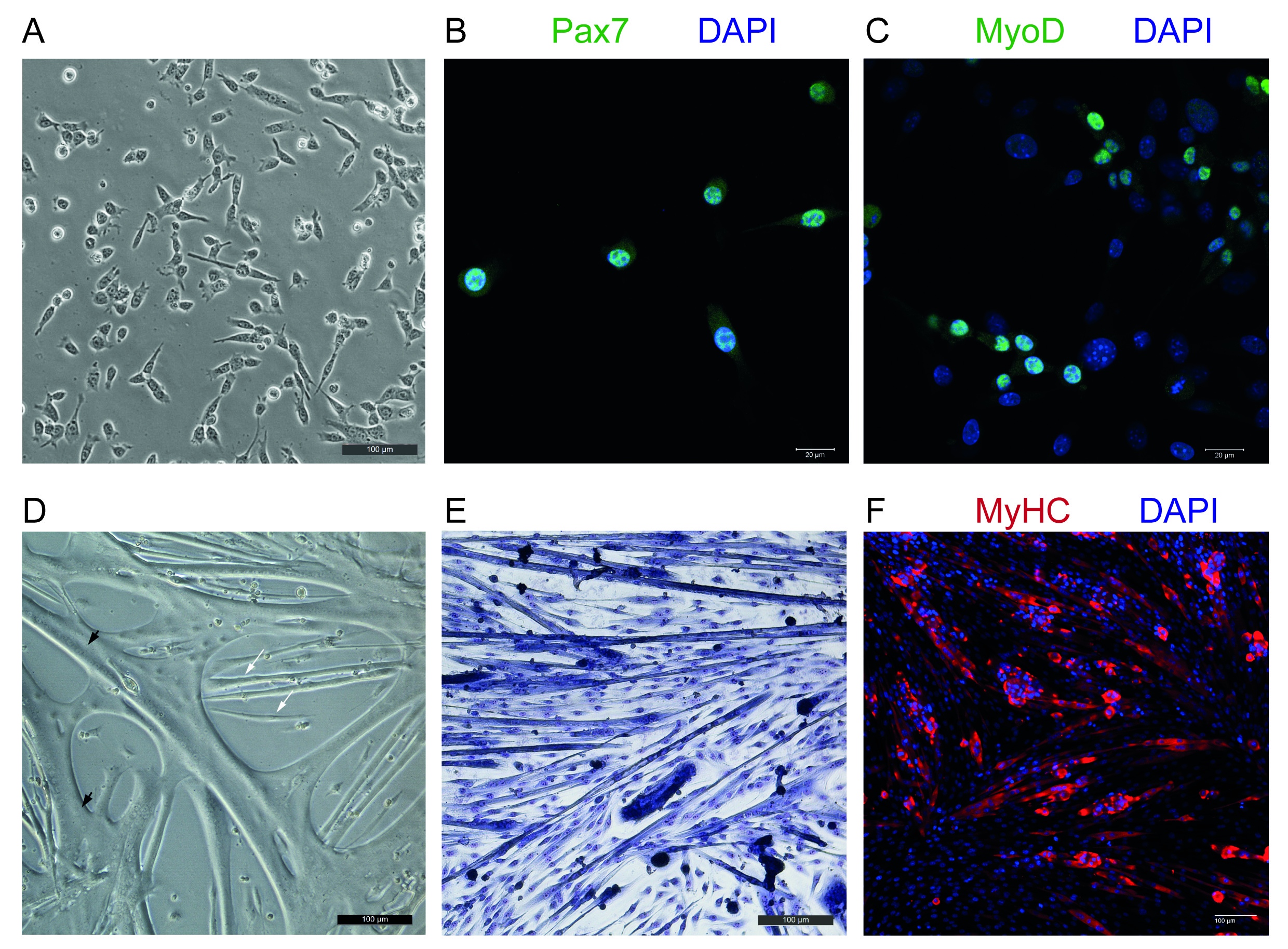

Supplement: Supplementary file 2 — Figure S2. Characterization of mouse primary satellite cells. (A) Undifferentiated SC at 60% confluency at passage 3. (B) SC are positive for Pax7 transcription factor. (C) MPC are a fraction of SC committed to myogenesis expressing MyoD transcription factor. (D) Multinucleated myotubes (black arrows) formed by fusion of SC (white arrows) at 7 days in differentiation culture conditions. (E) Fiber formation assay demonstrating long, multinucleated myotubes. Giemsa staining at 5 days in differentiation medium. (F) Myotubes express skeletal muscle-specific myosin heavy chain (MyHC). (JPG 3594 kb) [file 13287_2018_922_MOESM2_ESM.jpg]

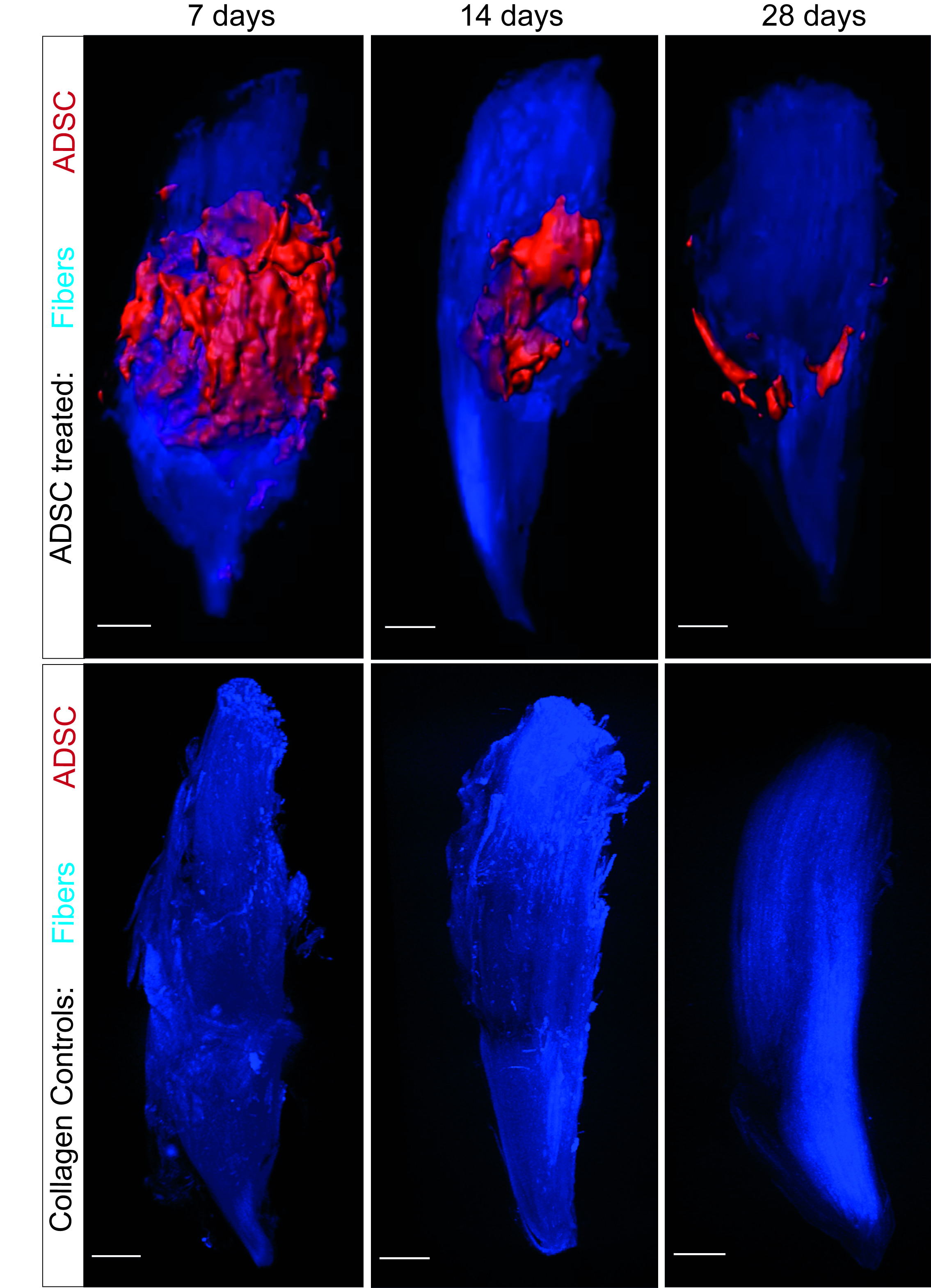

Supplement: Supplementary file 7 — Figure S4. OPT of single plane projection of the crushed TAs with implanted ADSC and collagen treated controls at 7, 14, and 28 days postimplantation. Blue: myofibers. Red: implanted ADSC. (JPG 2385 kb) [file 13287_2018_922_MOESM7_ESM.jpg]

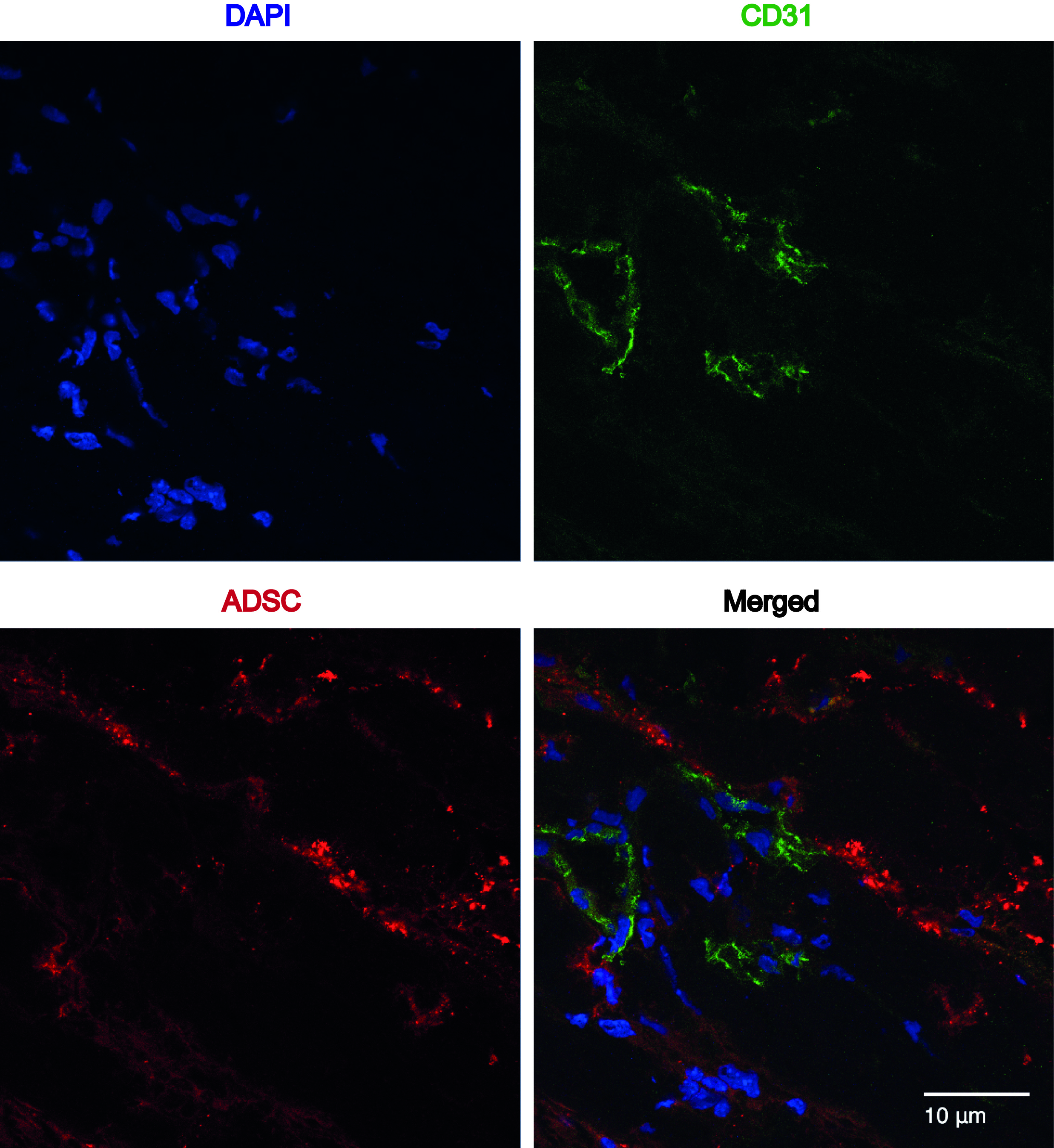

Supplement: Supplementary file 8 — Figure S3. ADSC do not differentiate into endothelial cells. Representative CD31 (green) staining showing that fluorescently red-labeled ADSC do not overlap with the endothelial cells in the TA muscle. Frozen sections of TA muscle were counterstained for cell nuclei (DAPI, blue). (JPG 5130 kb) [file 13287_2018_922_MOESM8_ESM.jpg]
